# Improving Palliative Care and Medical Assistance in Dying Practice in Canada: How Patients-Partners Could Contribute to Continuing Medical Education

**DOI:** 10.1089/pmr.2023.0008

**Published:** 2023-04-21

**Authors:** Jacynthe Rivest, Ghislaine Rouly, Marie-Josée Brouillette, Olivia Nguyen, Véronique Desbeaumes Jodoin

**Affiliations:** ^1^ Department of Psychiatry, Centre Hospitalier de l'Universite de Montreal (CHUM) and Centre de Recherche du CHUM (CRCHUM), Montreal, Québec, Canada.; ^2^ Centre of Excellence on Partnership with Patients and the Public, Montreal, Québec, Canada.; ^3^ Division of Infectious Diseases and Chronic Viral Illness Service, Department of Medicine, McGill University Health Centre, Montreal, Québec, Canada.; ^4^ Department of Psychiatry, McGill University Health Centre, Montreal, Québec, Canada.; ^5^ Palliative Care Division, CIUSSS du Nord-de-l'Île-de-Montréal, Montreal, Québec, Canada.; ^6^ Department of Family Medicine and Emergency Medicine, Université de Montréal, Montreal, Québec, Canada.

**Keywords:** continuing medical education, Medical Assistance in Dying, palliative care, patient engagement, psychological suffering

## Abstract

Medical Assistance in Dying (MAiD) is still considered an evolving practice in Canada. Practitioners are facing the challenge of staying up to date and hence need efficient continuing medical education (CME). A patient-partner has been recently invited as a keynote speaker to CME activities in Canada to share her perspectives and views about patient engagement in palliative care and MAiD practice, calling for compassion. To our knowledge, few data exist on patient-partners' contribution to CME on these topics. Based on that experience, we discuss different issues on patient engagement's contribution in such CME events and call for further research.

## Introduction

Living with advanced disease can be associated with physical pain, psychological distress, or decreased quality of life.^1^ Some might even express a wish to hasten death or request a physician-assisted death (PAD) when suffering.^2,3^ Medical practitioners frequently encounter these issues, especially in palliative care settings.^4^

PAD has become more accessible worldwide for the past decades. In Canada, the province of Quebec gave access to Medical Assistance in Dying (MAiD)^5^ in December 2015, as it became accessible across the rest of Canada in June 2016.^6,7^ MAiD was first an end-of-life option, but its accessibility has expanded, including to those whose death is not foreseeable.^8^ It is still an evolving practice. Health care professionals must then continuously adjust to changes in legal requirements and would like to be better informed about its relational, symbolic, psychological, and even spiritual aspects.^9^ Continuous medical education (CME) efforts must persist in addressing these gaps.

Patient partnership has been progressively implemented in health care with the purpose of improving quality of care, as patients are themselves experts of their experiences of illness.^10–13^ Patients-partners are frequently contributing to medical education^12^ and involved in CME events as well, although their specific contribution is underdocumented. Inspired from recent CME events, we discuss here about how patients-partners could potentially contribute to CME on MAiD and palliative care, highlighting opportunities and limits of such patient engagement.

## Patients-Partners' Contribution to Educational Challenges

Palliative care and MAiD practice are evolving, which make learning needs in that field almost constant. Outpatient palliative care is an example of a recent topic in CME, as multiple benefits from early palliative care have been well established.^14^ Growing educational needs are also seen in MAiD practice, as clinicians may encounter complex questions when exploring psychological suffering of requesters. Some may ask themselves whether the patient is suicidal or not, has a comorbid psychiatric disorder, or has lost capacity to consent.^6,15–18^ Preliminary data revealed that assessors are interested in further training on psychiatric aspects.^19^ Centering MAiD on the patient's needs remains a complex task.^20^

To address these educational challenges, experts in Canada are working on MAiD-specific trainings.^21^ For instance, two CME events involving patients-partners were also recently held in the province of Quebec. The psychiatric association of that province have organized in April 2021 a virtual half-day conference entitled *MAiD in Medicine and Surgery Settings: Which Role for the Psychiatrist*, since some of them are involved as a consultant in complex cases.^22–24^

Several topics were discussed, such as the prevalence of psychiatric comorbidities among requestors, or interventions for addressing suffering. Senior clinicians shared their field experienced with vignettes. More than 110 psychiatrists attended to this meeting. Another conference entitled “*Care without cure”: Psychiatry and Palliative Care, an Essential Partnership* was held in November 2021 by the University of Montreal. Thirteen speakers gave talks on many topics, including best practices in palliative care, treatment of depression at the end of life, and different perspectives on MAiD practice. About 100 health care professionals from various disciplines were attendees.

For both events, one patient-partner who has had accompanied a loved one through the dying process and had herself survived cancer was a keynote speaker. During her conference speech, she talked about the importance of meeting the needs of terminally ill. She called for preserving the dignity of MAiD requestors, and for compassion. She shared her experience, as a patient-partner, of being part of an interdisciplinary group responsible for guiding clinicians in their practice of MAiD in one tertiary care hospital. Feedback from participants for both events revealed a high satisfaction toward the patient-partner talk. Some even asked how they could recruit one in their own institution. Others reported being touched by the humanistic aspect of her testimony. It made us wonder if involving patients-partners in CME events should be considered a new standard. This question is relevant since innovations generate interest in CME.^25^

## Opportunities and Limits of Patient Engagement

Patients-partners are people with chronic illnesses playing an active role in various health care settings, medical education, quality improvement projects and governance of organizations.^26–29^ They benefit from a rigorous training program that prepares them to face clinical situations with adequate emotional distance, be mindful of confidentiality, and listen with empathy. They are considered full members of medical care and can sometimes provide direct care,^10^ including in palliative care settings. Offering the optimal quality of care in the end-of-life context often requires a deeper understanding of the patient's psychological experience of their illness.^30^ In that perspective, could involving patients-partners in CME on these topics help clinicians to better understand and stay patient-centered? Recent data indicate that MAiD programs that include real patients and their families might improve the quality of end-of-life care.^31,32^

It is important to acknowledge some potentials limitations. Although there is a long-standing culture on considering patient experience in CME events, the way that patients-partners should be prepared to do so are not documented. Institutions can find difficult to recruit such patients-partners. Some patients with a life-threatening condition might feel distressed when speaking about death. Death anxiety is an universal existential challenge among end-of-life patients.^33^ However, just like professionals, patients-partners are carefully selected and rigorously trained to be able to speak appropriately about illness and dying. How health care professionals experience collaborating with or listening to such patients remains unanswered. These limitations on best practices on patient engagement in CME warrant further investigation.

## Conclusion

As MAiD is an evolving practice in Canada, clinicians require continuous training to stay up to date. Any CME innovation that could help clinicians to learn about complex issues of palliative care and MAiD practice is welcome. The role of patient-partners is constantly growing in health care and medical education. Based on our recent CME experiences, we reflected on how patient engagement could become an interesting approach to address the multiple and complex aspects of palliative care and MAiD practice. More research is needed to clarify whether patients-partners' involvement now constitute a core component of continuing training in that field. Addressing such gaps through CME is essential to ensure patient-centered care.

## Abbreviations Used

CME: Continuing medical education
MAiD: Medical Assistance in Dying
PAD: physician-assisted death
